# Combination of *Panax ginseng* C. A. Mey and Febuxostat Boasted Cardioprotective Effects Against Doxorubicin-Induced Acute Cardiotoxicity in Rats

**DOI:** 10.3389/fphar.2022.905828

**Published:** 2022-06-22

**Authors:** Hayder M. Al-Kuraishy, Hany A. Al-Hussaniy, Ali I. Al-Gareeb, Walaa A. Negm, Aya H. El-Kadem, Gaber El-Saber Batiha, Nermeen N. Welson, Gomaa Mostafa-Hedeab, Ahmed H Qasem, Carlos Adam Conte-Junior

**Affiliations:** ^1^ Department of Clinical Pharmacology and Therapeutic, College of Medicine, Al-Mustansiriyah University, Baghdad, Iraq; ^2^ Department of Anesthesia, Al-Nukaba University College, Baghdad, Iraq; ^3^ Pharmacognosy Department, Faculty of Pharmacy, Tanta University, Tanta, Egypt; ^4^ Pharmacology and Toxicology Department, Faculty of Pharmacy, Tanta University, Tanta, Egypt; ^5^ Department of Pharmacology and Therapeutics, Faculty of Veterinary Medicine, Damanhour University, Damanhour, Egypt; ^6^ Department of Forensic Medicine and Clinical Toxicology, Faculty of Medicine, Beni-Suef University, Beni-Suef, Egypt; ^7^ Pharmacology Department & Health Research Unit, Medical College, Jouf University, Sakakah, Saudi Arabia; ^8^ Pharmacology Department, Faculty of Medicine, Beni-Suef University, Beni-Suef, Egypt; ^9^ Laboratory Medicine Department, Faculty of Applied Medical Sciences, Umm Al-Qura University, Makkah, Saudi Arabia; ^10^ Center for Food Analysis (NAL), Technological Development Support Laboratory (LADETEC), Federal University of Rio de Janeiro (UFRJ), Cidade Universitária, Rio de Janeiro, Brazil

**Keywords:** doxorubicin, *ginseng*, febuxostat, cardiac tropinin, BNP, TNF-α, glutathione peroxidase

## Abstract

Doxorubicin (DOX) is an anticancer agent for treating solid and soft tissue malignancies. However, the clinical use of DOX is restricted by cumulative, dose-dependent cardiotoxicity. Therefore, the present study aimed to assess the cardioprotective effects of *P. ginseng* C. A. Mey, febuxostat, and their combination against DOX-induced cardiotoxicity. Thirty-five Sprague Dawley male rats were used in this study. The animals were randomly divided into five groups, with seven rats per group. The control group received normal saline, the induced group received DOX only, and the treated group received *P. ginseng*, febuxostat, and their combination before DOX treatment. Biomarkers of acute cardiac toxicity were assessed in each group. Results showed that treatment with the combination of febuxostat and *P. ginseng* before DOX led to a significant improvement in the biomarkers of acute DOX-induced cardiotoxicity. In conclusion, the combination of *P. ginseng* and febuxostat produced more significant cardioprotective effects against DOX-induced cardiotoxicity when compared to either *P. ginseng* or febuxostat when used alone. The potential mechanism of this combination was mainly mediated by the anti-inflammatory and antioxidant effects of *P. ginseng* and febuxostat.

## Introduction

Doxorubicin (DOX) belongs to the anthracycline antibiotic family, considered the most effective anticancer agent for treating malignancies. However, the clinical use of DOX is restricted by its cumulative, dose-dependent cardiotoxicity, which may lead to irreversible heart failure or reduce the quality of life (Al-Kuraishy et al., 2015). In addition, DOX-induced cardiotoxicity is presented with acute heart failure, arrhythmias, and progressive cardiomyopathy (Al-Kuraishy and Al-Gareeb, 2016).

DOX-induced cardiotoxicity is mainly due to oxidative stress development, mitochondrial damage, and lipid peroxidation (Alkuraishy et al., 2017). DOX produces a variety of reactive oxygen species (ROS), which cause endoplasmic reticulum calcium leakage, DNA damage, and autophagy flux suppression, ultimately resulting in ferroptosis and lipid peroxidation (Abushouk et al., 2019; Podyacheva et al., 2021). The heart is sensitive to oxidative damage due to low antioxidant enzymes, large mitochondrial density/volume, and a higher oxygen consumption rate (Onohuean et al., 2021). Oxidative stress due to DOX-induced cardiotoxicity also develops due to the reduction of endogenous antioxidant capacity. Of interest, nuclear factor erythroid 2 (Nrf2), which acts as a sensor to regulate adaptive responses during oxidative stress, can potentially increase the expression of antioxidant enzymes. Nrf2 has been inhibited in DOX-induced cardiotoxicity (Xu et al., 2020).

Different herbal medicines and drugs alone or in combination have been tried to attenuate or prevent DOX-induced cardiotoxicity (Amin et al., 2021; Mu et al., 2021). Mainly, anti-inflammatory and antioxidant agents may play a crucial role in preventing DOX-induced cardiotoxicity (Sahasrabudhe et al., 2018). Several natural products demonstrated high potency against DOX-induced cardiotoxicity (El-Kharrag et al., 2017; Nurtay et al., 2021; Xie et al., 2021). Induced cardiotoxicity has been prevented by natural product nanoparticles (Al Shamsi et al., 2004; Amin and Mahmoud-Ghoneim 2011; Baig et al., 2019). For example, (Al Fatease et al., 2019) found that combinational polymeric micelles for delivery of resveratrol and quercetin in ovarian cancer were shown to be effective.

*P. ginseng* Family Araliaceae is found in eastern Asia and North America. *P. ginseng* contains more than 40 isolated active ingredients, including ginsenosides, sesquiterpenes, polyacetylenes, polysaccharides, and peptidoglycans (Farooqui et al., 2017). *P. ginseng* is increasingly used as alternative medicine or complementary medicine in treating different diseases, including cancer, neurodegenerative, cardiovascular, and chronic inflammation (Choi et al., 2015). *P. ginseng* reduces oxidative stress and restores antioxidant capacity in rats (Zhao et al., 2015). Its significant cardioprotective effects may lead to the synthesis of daily supplements that protect the heart from DOX-induced cardiotoxicity.

Febuxostat is a xanthine oxidase inhibitor indicated in patients with gout suffering from hyperuricemia, and it is used chiefly in the management of chronic gout (Zhang et al., 2021). With minimal adverse effects, Febuxostat is more effective than allopurinol at its standard doses (Wang et al., 2021).

The focus on xanthine oxidase inhibitors has increased due to their anti-inflammatory, antioxidant, and immune-modulatory effects, which might be beneficial in the treatment of different inflammatory diseases such as chronic obstructive pulmonary disease (COPD) and ulcerative colitis (El-Mahdy et al., 2020) and thus, it may be beneficial in alleviating oxidative stress and inflammation associated with DOX-induced cardiotoxicity. Therefore, the present study aimed to evaluate the possible cardioprotective effects of *P. ginseng* and febuxostat alone or in combination against DOX-induced cardiotoxicity.

## Materials and Methods

### Drugs and Chemicals

Doxorubicin (Adricin^®^) was obtained from Hikma Pharmaceuticals (Cairo, Egypt). Febuxostat 120 mg (Feburic^®^) from Alhikma Co. (Jordan) was used. The commercially used *P. ginseng* was a well-prepared capsule (*P. ginseng* capsule, Euro Herbs, California Gold Nutrition, United States). It was prepared from the plant’s roots with the following botanical characteristics (phylum: Embryophyta Siphonogama, subphylum: Angiospermae, class: Dicotyledoneae, subclass: Archichlamydeae, order: Umbelliflorae, family: Araliaceae, genus: *Panax*). All the other chemicals were of high analytical grade and were commercially obtained (Global Medical Instrumentation, Ohaos, United States). DOX, *P. ginseng,* and febuxostat were dissolved in normal saline separately. The dose of each drug was calculated according to previous experimental studies in which these doses exhibited the best results (Ammar et al., 2011; Shamim and Khan, 2018; Abdel-Aziz et al., 2020).

### Animals

Thirty-five Sprague Dawley male rats were used in this study. The animals were obtained from the Iraqi Center for Cancer and Medical Genetic Research (Mustansiriyah University, Iraq). Their body weights ranged from 150 to 250 g. The rats were housed in sterile cages and kept at 25°C with a 12/12 light-dark cycle. The rats were allowed to chow pellets and drink tap water *ad libitum* (standard pellet, Purina, United States). They were left for 2 weeks without interference to acclimatize. All cages and materials used to prepare food were sterile; all rats were free of any illness during the observation period. The used animal procedures were held according to the guide for the care of laboratory animals (LaFollette et al., 2020). The experiment was carried out in accordance with the criteria for the care and use of laboratory animals, which were authorized by the Research Ethical Committee (Faculty of Pharmacy, Tanta University, Egypt), Approval No. (PO-2021-00126-E).

### Study Design

After 2 weeks of acclimatization, one diseased rat was excluded. The animals were randomly divided into five groups, with seven rats in each group. On day ten, all groups were sacrificed. Control group: Received normal saline per oral (2.5 ml/kg)/day) for 10 days (*n* = 7), Doxorubicin group: Received normal saline per oral (2.5 ml/kg/day) for 10 days, followed by a single dose of DOX (15 mg/kg) intraperitoneally (IP) (Ammar et al., 2011) and on day eight, serving as a DOX group (*n* = 7). DOX + *P. ginseng* group: Received *P. ginseng* per oral (100 mg/kg) (Shamim and Khan, 2018) daily for ten successive days, and on day eight, 1 h after drug administration, a single dose of DOX (15 mg/kg) IP, was given (*n* = 7). DOX + febuxostat group: Received febuxostat (10 mg/kg) (Abdel-Aziz et al., 2020) per oral daily for ten successive days, and on day eight, 1 h after drug administration, a single dose of DOX (15 mg/kg) IP was given (*n* = 7). DOX + combination group: Received *P. ginseng* (100 mg/kg) and febuxostat (10 mg/kg) per oral, 1 h apart for successful separation and to avoid physical drug interaction, daily for 10 days, and on day eight, 1 h after drug administration, a single dose of DOX (15 mg/kg) IP was given (*n* = 7). On the 11th day of the study, animals were sacrificed, and hearts were taken for histopathological observations. Blood samples were taken for biochemical analysis.

### Samples Collection

At room temperature, 25°C, the rats were anesthetized using chloroform. Blood samples were collected by intracardiac puncture in sterile, labeled tubes and then centrifuged for 10 min at 3,000 rpm. The samples were stored at −20°C to be assessed later. The rats were sacrificed to obtain the hearts immediately immersed in normal iced saline to prevent ischemic heart injury caused by further beating. Hearts were fixed in neutral buffered formalin (10%) to harden the tissue and avoid structural changes due to autolysis by the tissue lysosomal enzymes.

### Assessment of Biochemical Variables

Serum levels of brain natriuretic peptide (BNP), cardiac troponin-I (cTn-I), caspase-3, glutathione peroxidase (GP), malondialdehyde (MDA), lipid peroxidase (LPO) and tumor necrosis factor-alpha (TNF-α) were determined using ELISA kit methods (MyBioSource, San Diego, CA, United States).

### Histopathological Studies

Animals’ hearts were fixed in a formaldehyde solution (10%) to harden the tissue. Cross-sectional cuts were made to obtain the ventricles. Dehydration was done gradually to prevent shrinkage of the tissue. Then an infiltration process was done to support the tissue during the sectioning step by filling the tissue with paraffin. They were embedded in the following sequence: distilled water for washing, 70% alcohol for 2 h, 80% alcohol for 2 h, 90% alcohol for 2 h, 95% alcohol for 2 h, 100% alcohol for 2 h, xylene for 1 h, and finally paraffin for 2 h. The tissue is embedded and solidified into a hard paraffin cube at room temperature and then sectioned by a microtome to produce a thin tissue section of a known thickness. The sections were placed on the slides and left to dry for about 24 h. Finally, the slides were stained with two different dyes (eosin and hematoxylin).

### Statistical Analysis

Data are presented as mean ± S.D. (standard deviation) Multiple comparisons among different groups were performed by one-way analysis of variance (ANOVA), followed by Tukey-Kramer as a posthoc test using GraphPad Prism version 9 (GraphPad Software, Inc. San Diego, CA, United States). Results were considered statistically significant at *p* < 0.05.

## Results

### Biochemical Parameters

DOX-induced cardiotoxicity was evidenced by the significant reduction in GP serum level and the increase in LPO and MDA levels compared to the control (*p* < 0.05). Febuxostat administration before DOX led to a significant elevation of GP serum levels (*p* < 0.05). However, when compared to the DOX group, the suppressive effect of febuxostat on LPO and MDA was insignificant (*p* > 0.05). Also, *P. ginseng* administration before DOX significantly elevated the GP and suppressed the MDA levels (*p* < 0.05) but the suppressive effect on LPO was insignificant (*p* > 0.05). Moreover, the combination of febuxostat and *P. ginseng* demonstrated a significant improvement in GP, LPO, and MDA (*p* < 0.05) (Table 1).

**TABLE 1:** Effects of *P. ginseng* and Febuxostat on oxidative stress biomarkers in DOX-induced cardiotoxicity.

Parameters	Control (*n* = 7)	Doxorubicin (*n* = 7)	DOX + Febuxostat (*n* = 7)	DOX + *P. ginseng* (*n* = 7)	DOX + Combination (*n* = 7)
GP (pmol/L)	24.83 ± 1.97	14.5 ± 2.32^a^	19.2 ± 1.34^b^	19 ± 0.83^b^	24 ± 3.82^b^ ^,^ ^c^ ^,^ ^d^
LPO (nmol/L)	14.83 ± 1.72	26.17 ± 2.83^a^	24.6 ± 1.23	24.7 ± 0.77	16.75 ± 2.03^b^ ^,^ ^c^ ^,^ ^d^
MDA (nmol/L)	1.1 ± 0.21	1.93 ± 0.34^a^	1.60 ± 0.10	1.51 ± 0.15^b^	1.175 + 0.219^b^ ^,^ ^c^

Results are expressed as mean ± SD; *p < 0.05, BNP, brain natriuretic peptide; GSH: GP, Glutathione peroxidase; LPO, Lipid peroxide; MDA, Malondialdehyde. Significant difference vs.

a Represents the control.

b Represents the Doxorubicin group.

c Represents the DOX + Febuxostat group.

d Represents the DOX + *P. ginseng* group.

**FIGURE 1:**
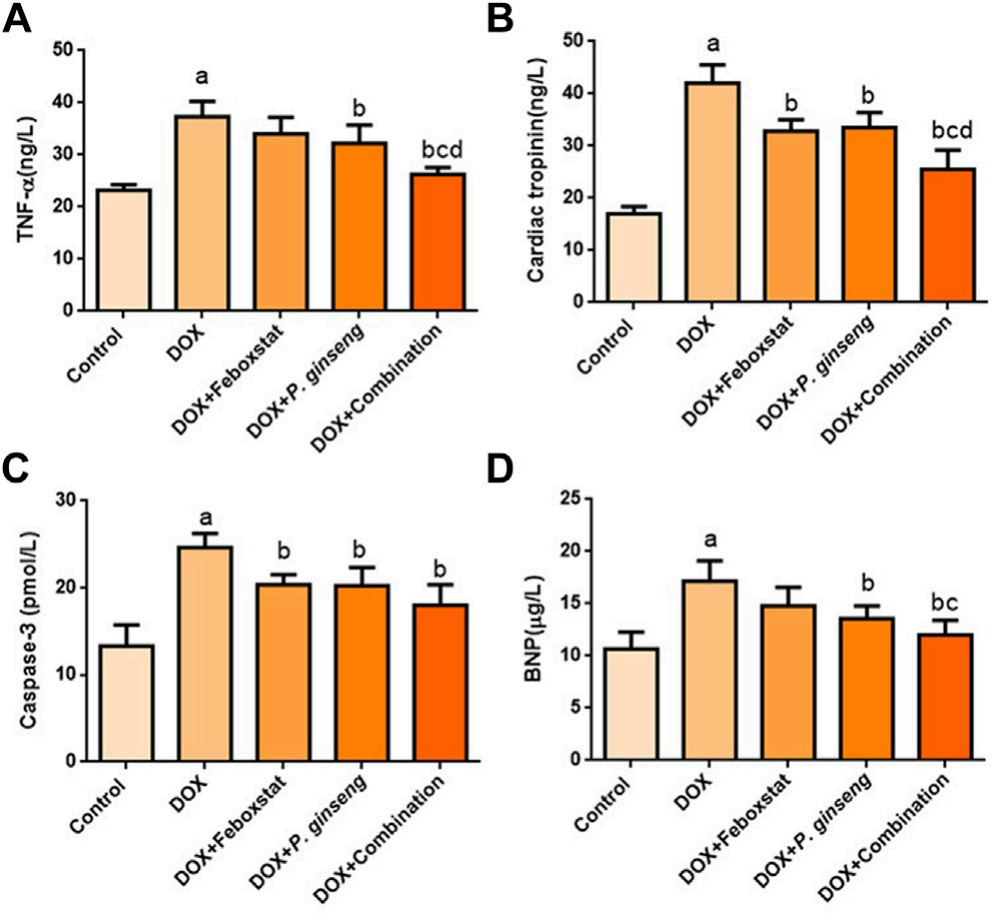
Effects of drug treatments on **(A)** Serum TNF-α level, **(B)** Cardiac Tropinin level **(C)** Serum Caspase-3 level, and **(D)** Serum BNP level.

DOX administration significantly increased cTnI, BNP, caspase-3, and TNF-α levels (*p* < 0.05). Febuxostat administration before DOX led to a significant decrease in cTnI and caspase-3 (*p* < 0.05), but the effect on BNP and TNF-α was insignificant (*p* > 0.05) when compared to the DOX group. Treatment with *P. ginseng* led to a significant decrease in cTnI, BNP, caspase-3, and TNF-α (*p* < 0.05). Furthermore, the combination of febuxostat and *P. ginseng* illustrated a significant suppressive effect on TNF-α and cTnI as compared to each single-drug therapy (*p* < 0.05) (Figure 1).

### Histopathological Changes

**FIGURE 2:**
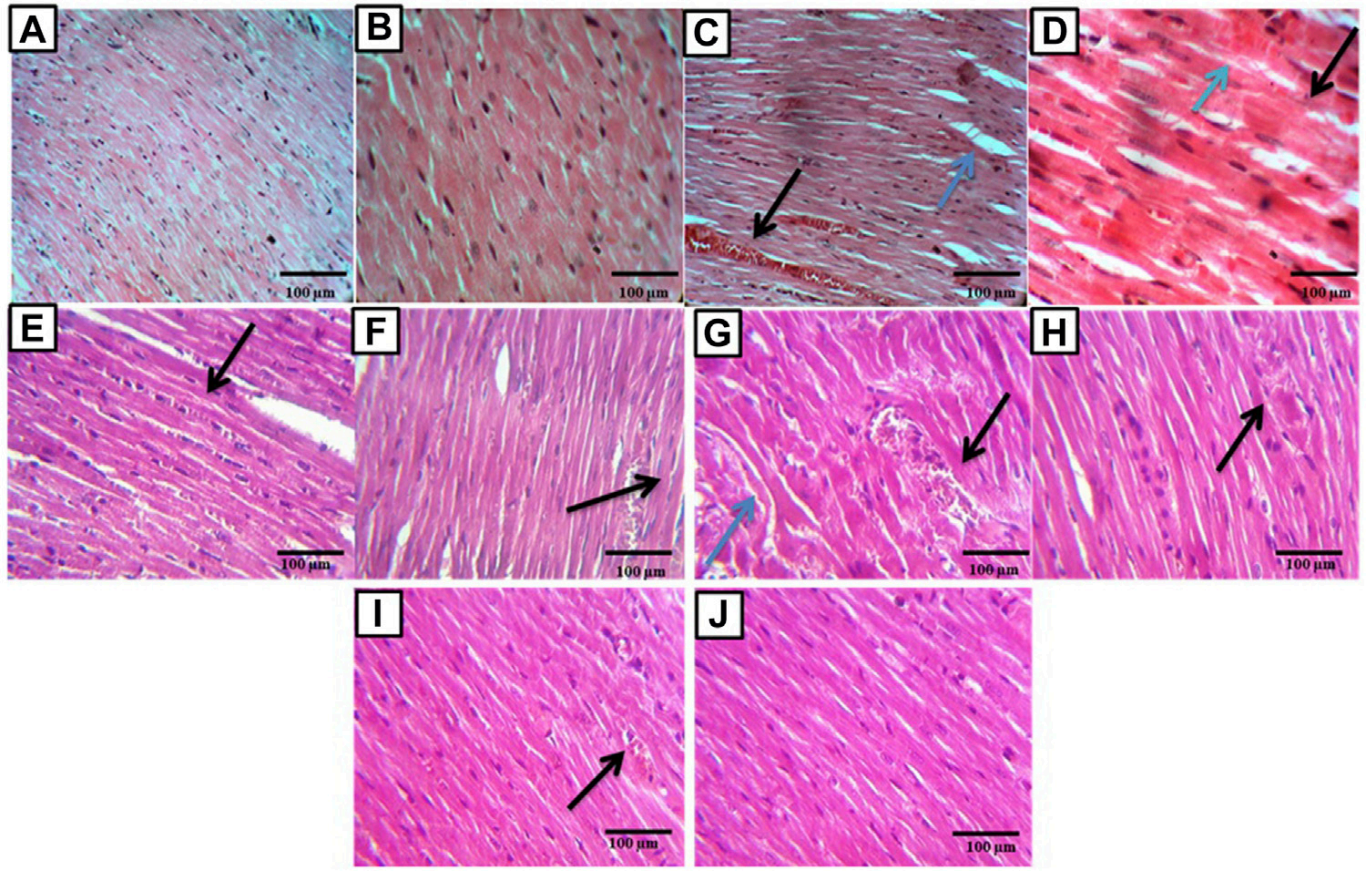
Histopathological examination of cardiac sections: **(A,B)** sections showed normal rat myocardial tissue, magnification ×40, ×100 respectively. **(C)** Section of the DOX affected myocardial tissue showed congested and dilated blood vessel (black arrow) with edema (blue arrow) (×40). **(D)** Section of the DOX affected myocardial tissue showed fragmented muscle fibers (black arrow), decreased number of nuclei, and extravasation of R.B.C.s (blue arrow) (×100). **(E,F)** Sections of the DOX + Febuoxatat group showed improved DOX-induced myocardial damage with preserved nuclei and no muscle fibers fragmentation but congested and dilated blood vessels are still present (arrow) (×40), no muscles fibers fragmentation but odema and extravasation of R.B.C.s are still present (black arrow) (×100). **(G,H)** Sections of the DOX + *P.Ginseng* group showed improved DOX-induced myocardial damage apart from odema and vaculations (blue arrows) (×40) and showed area of coagulative necrosis, vascular congestion and chronic inflammation cell (black arrow) (×100). **(I,J)** sections of the DOX + combination group showed nearly normal-looking cardiac muscle tissue (×40) apart from a congested blood vessel (black arrow) (×100).

The control group sections showed a normal myocardial tissue structure with a peripherally located normal oval nucleus and branching striated muscle fibers**.** While the sections of the DOX group showed many congested vessels with extravasation of red blood cells, edema, cytoplasmic vacuolations, decreased nuclei, loss of muscle fiber striation, and fragmentation with necrosis. While sections of the febuxostat group showed improved myocardial damage with preserved nuclei and without fragmentation of muscle fibers, congested and dilated, blood vessels were still present. Also, edema and extravasation of red blood cells were still present. The *P. ginseng* group myocardial tissue section showed improved myocardial damage apart from edema and vacuolations. In addition, sections of the combination group showed nearly normal-looking cardiac muscle tissue. Light microscopic magnification was done using two powers, ×40 and ×100 (Figure 2).

## Discussion

Cardiac toxicity is the principal dose-limiting factor for DOX use as an anticancer treatment (Yin and Shen, 2021). DOX-induced cardiotoxicity develops due to complex molecular mechanisms including mitochondrial dysfunction, apoptosis, necrosis, and oxidative stress (Cardinale et al., 2020). cTn-I has been regarded as the gold standard biomarker for myocardial injury and cardiotoxicity (Murabito et al., 2020; Abdalla et al., 2021). Of note, cTn-I is released into the plasma when cardiac myocytes are injured (Varricchi et al., 2018).

The present study clearly showed that DOX-induced myocardial injury led to significant elevations in the cTn-I and LPO serum levels, as consistent with recent findings (Kumari et al., 2020; Lan et al., 2020). The increase in LPO levels could be attributed to the toxic effects of DOX on the cardiomyocytes through oxidative degradation. Febuxostat reduced cTn-I and LPO serum levels, as documented by recent studies (Ayza et al., 2020; Tanaka et al., 2021). For this reason, febuxostat may exert a cardioprotective effect against DOX-induced cardiotoxicity.

Regarding the effect on oxidative stress, DOX showed a significant decline in the endogenous antioxidant capacity, as evident by a significant reduction in GP serum levels and a remarkable increase in MDA levels, confirming the well-known hypothesis that free radicals play a significant role in DOX-induced cardiotoxicity, and these results were in line with previous reports (Gammella et al., 2014; Lenihan, 2014; Nebigil and Désaubry, 2018). Moreover, the pretreatment with febuxostat led to a significant increase in GP levels and a substantial decrease in MDA serum, coinciding with different studies (Shim et al., 2017; Ahmed et al., 2021).

Myocardial TNF-α was an autocrine contributor to myocardial dysfunction and cardiomyocyte death in ischemia-reperfusion injury, sepsis, and chronic heart failure (Sheppard et al., 2013). In the present study, DOX significantly elevated the TNF-α serum level. However, febuxostat lowered the TNF-α serum level. This outcome corresponds with different studies that disclosed a potential effect of febuxostat in reducing pro-inflammatory cytokines (El-mahdy et al., 2020; Ahmed et al., 2021). Likewise, BNP was significantly increased with DOX treatment. The results of the present study are in agreement with previous results (Sheppard et al., 2013). On the other hand, another study reported that low BNP serum levels during acute DOX-induced cardiotoxicity might be due to inhibiting the expression of the BNP gene (Murabito et al., 2020).

In the present study, febuxostat showed a reasonable but non-significant decrease in BNP serum levels, as shown previously (Ahmed et al., 2021). This was due to the antioxidant, anti-inflammatory, and anti-apoptotic effects of febuxostat (Al-Kuraishy et al., 2019). Furthermore, DOX may induce myocardial cell apoptosis by activating mitochondrial caspase-3 (Fidale et al., 2018). This study clearly showed a significant elevation in the plasma level of caspase-3 with DOX treatment, as revealed previously (Shim et al., 2017). However, febuxostat pretreatment significantly decreased caspase-3 serum levels, coinciding with the findings of a previous study (Krishnamurthy et al., 2015).

Our observations demonstrated that febuxostat could limit the infarct size of acute myocardial infarction. This reduction is accompanied by changes in the level of matrix metalloproteinase enzymes, biomarkers of cardiac cell injury (Al-Kuraishy et al., 2019).

The present study also showed that cTn-I was reduced significantly with *P. ginseng* pretreatment, coinciding with a previous experimental study (Al Shamsi et al., 2006), which showed that the active ingredient of the *P. ginseng* extract, ginsenoside-Rg1, enhanced angiogenesis and reduced ventricular remodeling in a rat model of myocardial infarction. Similarly, *P. ginseng* pretreatment showed a significant decrease in serum BNP and caspase-3 levels, coinciding with various studies (Mohan et al., 2018; Hamza et al., 2021; Murali et al., 2021).

Moreover, *P. ginseng* significantly elevated the GP serum level. Several studies (Al-Dabbagh et al., 2018; Li et al., 2020; Nazarbek et al., 2021) confirmed this finding. As well, the MDA serum level showed a significant reduction after the administration of *P. ginseng*, coinciding with a previous report (Wang et al., 2019). Furthermore, *P. ginseng* reduced the TNF-α serum level, which might be attributed to TNF-α inhibition in the myocardium (Parlakpinar et al., 2019).

The oxidative degradation of lipids was also ameliorated by the antioxidant effect of *P. ginseng*. In this study, *P. ginseng* pretreatment showed a decline in the LPO level. This result corresponds with previous studies confirming the antioxidant effect of *P. ginseng* (Zhang et al., 2018; Benassi et al., 2021). Indeed, ginsenoside-Rg1 enhanced angiogenesis by suppressing the progression of cardiac fibrosis (Geng et al., 2020; Abdalla et al., 2021). Thus, the remarkable cardioprotective effect of *P. ginseng* in the present study may be produced by its anti-proliferative action, as evident by the amelioration of DOX-induced histopathological changes (Yu et al., 2021).

The potential protective mechanism of *P. ginseng* against DOX-induced cardiotoxicity is related to the antioxidant, anti-apoptotic, and anti-inflammatory effects of *P. ginseng* constituents. An experimental study demonstrated that *P. ginseng* mitigated the electrocardiographic and histopathological changes induced by DOX and restored the antioxidant capacity (Chen et al., 2021). Another study reported that *P. ginseng* constituents might be a novel candidate for improving DOX-induced cardiotoxicity (Wan et al., 2021).

Of interest, the pretreatment with a combination of *P. ginseng* and febuxostat significantly reduced the biomarkers of DOX-induced cardiotoxicity more than either *P. ginseng* or febuxostat when used alone. Previous studies declared that both *P. ginseng* and febuxostat had antioxidant and anti-inflammatory properties that were interrelated at the molecular level. Both *P. ginseng* and febuxostat exerted antioxidant properties partially by interfering with NADPH oxidase activity (Lu et al., 2018; Juaid et al., 2021). Additionally, both *P. ginseng* and febuxostat had anti-inflammatory and antioxidant properties that significantly reduced oxidative stress and inflammatory reactions (Sung et al., 2021; Xu et al., 2021).

Taken together, the present study confirmed the protective effects of the febuxostat and *P. ginseng* combination against DOX-induced cardiotoxicity as manifested by the considerable improvement in toxicity biomarkers and histological damage.

Applying the current findings on clinical studies could be of a great importance in the reduction of DOX-induced cardiotoxicity in DOX-treated patients as in leukemia, lymphoma, and solid tumors (Kalyanaraman, 2020). Anti-inflammatory and antioxidant agents could be a therapeutic potential strategy in the mitigation of cardiotoxicity in patients treated by DOX (Bruynzeel et al., 2007). Therefore, *P. ginseng* and febuxostat in virtue of their antioxidant and anti-inflammatory properties could be a novel combination in reducing DOX-induced cardiotoxicity in high-risk patients.

## Conclusion

The current study indicated that the combination of *P. ginseng* and febuxostat confers better cardioprotective effects against DOX-induced cardiotoxicity than single-drug therapy. The anti-inflammatory and anti-apoptotic activities may mediate the potential cardioprotective effects and antioxidant effects of *P. ginseng* and febuxostat. Therefore, preclinical and clinical studies are warranted to confirm the clinical benefit of *P. ginseng* or febuxostat in patients at high risk of developing DOX-induced cardiotoxicity.

## Data Availability

The raw data supporting the conclusion of this article will be made available by the authors upon reasonable request.
